# Dietary Approaches in the Management of Diabetic Patients with Kidney Disease

**DOI:** 10.3390/nu9080824

**Published:** 2017-07-31

**Authors:** Gang Jee Ko, Kamyar Kalantar-Zadeh, Jordi Goldstein-Fuchs, Connie M. Rhee

**Affiliations:** 1 Harold Simmons Center for Kidney Disease Research and Epidemiology, Division of Nephrology and Hypertension, University of California Irvine, School of Medicine, Orange, CA 92868, USA; lovesba@gmail.com (G.J.K.); kkz@uci.edu (K.K.-Z.); 2 Department of Internal Medicine, Korea University, School of Medicine, Seoul 08308, Korea; 3 Department of Medicine, Tibor Rubin Veteran Affairs Health System, Long Beach, CA 90822, USA; 4 Los Angeles Biomedical Research Institute at Harbor-UCLA, Torrance, CA 90502, USA; 5 Sierra Nevada Nephrology Consultants, Reno, NV 89511, USA; Djordijrn@sbcglobal.net; 6 Department of Internal Medicine, University of Nevada Reno, School of Medicine, Reno, NV 89557, USA

**Keywords:** diabetes, kidney disease, nutrition, diet

## Abstract

Chronic kidney disease (CKD) is one of the most prevalent complications of diabetes, and patients with diabetic kidney disease (DKD) have a substantially higher risk of cardiovascular disease and death compared to their non-diabetic CKD counterparts. In addition to pharmacologic management strategies, nutritional and dietary interventions in DKD are an essential aspect of management with the potential for ameliorating kidney function decline and preventing the development of other end-organ complications. Among DKD patients with non-dialysis dependent CKD, expert panels recommend lower dietary protein intake of 0.8 g/kg of body weight/day, while higher dietary protein intake (>1.2 g/kg of body weight/day) is advised among diabetic end-stage renal disease patients receiving maintenance dialysis to counteract protein catabolism, dialysate amino acid and protein losses, and protein-energy wasting. Carbohydrates from sugars should be limited to less than 10% of energy intake, and it is also suggested that higher polyunsaturated and monounsaturated fat consumption in lieu of saturated fatty acids, trans-fat, and cholesterol are associated with more favorable outcomes. While guidelines recommend dietary sodium restriction to less than 1.5–2.3 g/day, excessively low sodium intake may be associated with hyponatremia as well as impaired glucose metabolism and insulin sensitivity. As patients with advanced DKD progressing to end-stage renal disease may be prone to the “burnt-out diabetes” phenomenon (i.e., spontaneous resolution of hypoglycemia and frequent hypoglycemic episodes), further studies in this population are particularly needed to determine the safety and efficacy of dietary restrictions in this population.

## 1. Introduction

Epidemiologic data show that the prevalence of diabetes is increasing worldwide, particularly in the United States (US) where 29.1 million people (9.3% of the population) are estimated to have diabetes [1]. While longitudinal data from the US show that the incidence of diabetic end-organ complications has declined over the past two decades [2], diabetes remains a major source of morbidity and mortality due to its staggering rise in prevalence [3]. Indeed, United States Renal Data System (USRDS) registry data show that diabetes accounts for nearly half of the end-stage renal disease (ESRD) population in the US [4]. Thus, two major goals in the management of diabetic kidney disease (DKD) are stabilization of kidney function and prevention of the development of other end-organ complications. Provision of balanced nutritional therapy, in conjunction with pharmacologic interventions that optimize glycemic status, lipid levels, and blood pressure, are cornerstones of the management of DKD patients [5]. In this review, we summarize current clinical practice guidelines and supporting evidence regarding the nutritional management of patients with DKD.

## 2. Clinical Practice Guidelines on the Nutritional Management of Diabetic Kidney Disease

**Figure 1:**
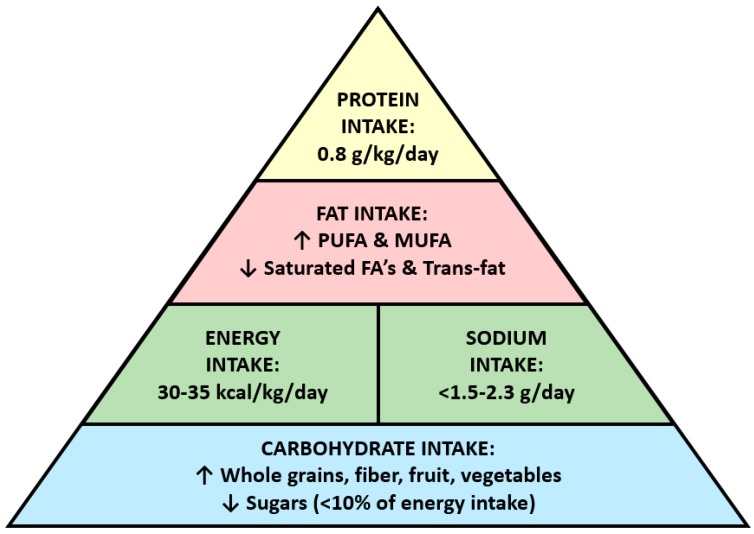
Diabetic Kidney Disease Food Pyramid. Abbreviations: PUFA, polyunsaturated fatty acids; MUFA, monounsaturated fatty acids; FA, fatty acid.

**Figure 2:**
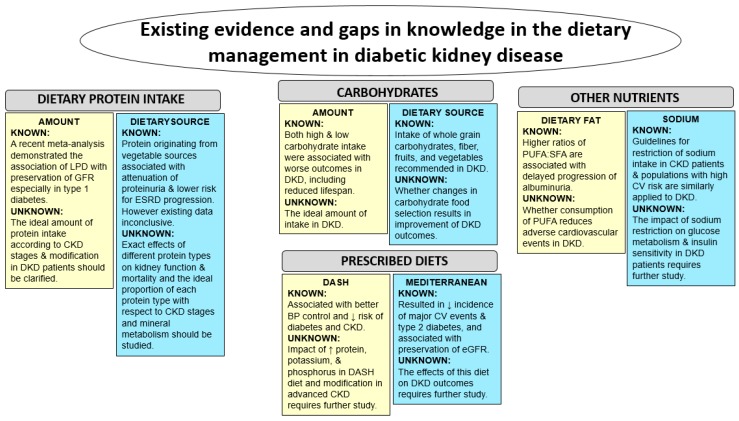
Summary of existing evidence and gaps in knowledge in the dietary management of diabetic kidney disease. Abbreviations: LPD, low protein diet; GFR, glomerular filtration rate; CKD, chronic kidney disease; DKD, diabetic kidney disease; ESRD, end-stage renal disease; CV, cardiovascular; PUFA, polyunsaturated fatty acids; SFA, saturated fatty acids.

While nutritional therapy is an essential aspect of the treatment of DKD, current clinical practice guidelines (Figure 1) do not wholly address all areas of dietary management due to existing controversies and gaps in knowledge about particular interventions (e.g., dietary protein restriction) in this population (Figure 2). Furthermore, recommendations may differ across the stages of DKD due to differential risk-to-benefit ratio profiles of certain management strategies across varying levels of kidney function. For example, among advanced chronic kidney disease (CKD) and ESRD patients who may be susceptible to hypoglycemia, intensive glycemic control may be associated with heightened risk of adverse outcomes [6,7]. In 2007, the National Kidney Foundation (NKF) Kidney Disease Outcome Quality Initiative (KDOQI) Clinical Practice Guidelines for the Nutritional Management of Diabetes and CKD was first introduced [8]. These seminal guidelines were primarily focused upon recommending dietary protein restriction among DKD patients, with a specific target dietary protein intake of 0.8 g/kg of body weight (BW)/day (approximately 10% of total caloric intake) among those with stages 1–4 CKD based on moderate to strong evidence. A similar management strategy for DKD patients with stage 5 CKD was also advised. However, in 2012, a Kidney Disease Improving Global Outcome (KDIGO) expert panel recommended comparatively liberal parameters with respect to dietary protein intake, advising that it should be maintained at 0.8 g/kg BW/day while avoiding levels above 1.3 g/kg BW/day [9]. KDIGO guidelines also highlighted that there was insufficient evidence demonstrating that long term restriction of dietary protein intake below 0.8 g/kg BW/day is beneficial in DKD patients. Other experts suggest a low protein intake of 0.6 to 0.8 g/kg/day including 25% to 50% of high biologic value protein as a more effective dietary strategy [10,11].

In 2014, a consensus conference for DKD convened by the American Diabetes Association (ADA) in collaboration with the American Society of Nephrology (ASN) and the National Kidney Foundation (NKF) summarized existing evidence and issued updated recommendations regarding the optimal intake of various macro-nutrients. With respect to dietary protein intake, the expert panel recommended a “usual level of dietary protein intake” among DKD patients of approximately 16−18% of total caloric intake [12]. The panel′s recommendations regarding optimal intake of other macro-nutrients are summarized in Table 1.

## 3. Dietary Protein Intake in Diabetic Kidney Disease: Non-Dialysis Dependent Chronic Kidney Disease

### 3.1. Quantity of Dietary Protein Intake

Dietary protein requirements among diabetic patients without kidney disease are considered to be equivalent to that of the general population. In the general population, National Health and Nutritional Examination Survey (NHANES) data demonstrated that the average dietary protein intake of US adults is 1.34 g/kg of ideal body weight (IBW)/day or 1.09 g/kg of actual body weight (ABW)/day, which substantially exceeds the requisite amount needed to avoid negative nitrogen balance for normal healthy adults (i.e., 0.8 g/kg ABW/day) [9,13]. When examined according to demographic characteristics, higher dietary protein intake was observed among men vs. women (1.36 vs. 1.25 g/kg IBW/day, respectively); Mexican-American and Latino participants vs. other racial/ethnic groups (1.43, 1.24, 1.30, and 1.35 g/kg IBW/day among Mexican-American/Latino, non-Hispanic Black, non-Hispanic White, and other racial/ethnic groups, respectively). Incrementally higher dietary protein intake was also observed in younger vs. older aged participants (1.40, 1.38, 1.32, 1.22, 1.16, and 1.08 g/kg IBW/day for participants 20–34, 35–44, 45–54, 55–64, 65–74, and ≥74 years of age, respectively). However, little is known about how dietary protein intake varies among diabetic vs. non-diabetic patients in the broader US population.

It should be noted that type 2 diabetic patients may be more frequently exposed to protein-rich food sources as a means (1) to promote weight loss, such as fad dietary regimens including the Atkins or Protein Power diets; or (2) to avoid postprandial hyperglycemia by replacing carbohydrate-rich foods with protein-rich foods [8,14]. However, receipt of these high protein diets may heighten diabetic patients′ risk of developing impaired renal function [15]. In contrast to fats and carbohydrates, high dietary protein intake increases glomerular filtration rates (GFRs) in order to excrete protein-derived nitrogen metabolites [16,17,18], as shown in a recent meta-analysis of 30 randomized controlled trials (RCTs) that included 2160 participants [19]. Renal hemodynamic changes induced by excess protein intake may also exert deleterious consequences over time. For example, a population-based study of patients with mild kidney dysfunction (estimated GFR (eGFR) 55–80 mL/min/1.73 m^2^) who had long-term consumption of a high protein diet (20% versus 10% of total daily calories) showed a significant decline in kidney function over an 11-year follow up period [20]. Although current nutritional guidelines for diabetic patients without kidney disease have not recommended dietary protein restriction [21], avoidance of excess dietary protein intake may be beneficial in preventing long-term renal complications.

Among patients with DKD, the potential benefits and risks of a low protein diet (LPD) have been widely debated. Many studies, including RCTs, have shown a beneficial impact of LPD upon trajectory of kidney function in this population [22,23,24,25,26]. More specifically, it has been suggested that a LPD, often used in conjunction with essential amino acids and ketoacids, may reduce proteinuria, uremic burden, metabolic derangements (e.g., metabolic acidosis), and oxidative stress leading to attenuation in the progression of CKD and delayed initiation of dialysis treatment [11,27,28]. Conversely, other studies have not confirmed that a LPD favorably impacts CKD trajectory [29,30,31]. A Cochrane review of 12 RCTs concluded that administration of LPDs showed a small but non-significant benefit upon slowing of eGFR decline [32]. It should be noted that the study populations’ sizes and stages of CKD, as well as durations of treatment intervention were highly variable across studies, and adherence to dietary recommendations were not systematically assessed (i.e., actual protein intake may have differed from prescribed intake). In a meta-analysis of 13 RCTs, LPD was associated with significant improvement in eGFR (5.82 (95% CI) (2.30 to 9.33) mL/min/1.73 m^2^) [33]. These beneficial effects were observed independent of the type of diabetes, stage of CKD, and duration of the intervention. However, in subgroup analyses that assessed dietary compliance using urinary urea excretion, improvement in eGFR was observed only among those in whom compliance was fair. Notably, another meta-analysis has shown a differential effect of dietary protein restriction upon CKD progression according to type of diabetes, such that restriction was beneficial in type 1 diabetic patients but not in those with type 2 diabetes [34]. However, it should be noted that the meta-analysis had proportionately fewer studies of type 2 diabetes patients. Another prospective study of type 2 diabetic patients has demonstrated that a LPD was beneficial upon kidney function and proteinuria trajectory over a three-year period [35].

### 3.2. Sources of Dietary Protein Intake

In addition to quantity, the quality and characteristics of dietary protein intake and their impact upon kidney function have also been a focus of investigation in the broader CKD population. In an RCT with follow up over four years, soy protein consumption as a replacement for animal sources of protein (35% animal, 35% soy-origin, and 30% vegetable proteins) was associated with significant improvement in proteinuria as compared with usual protein consumption (70% animal and 30% vegetable proteins) [36]. A recent prospective observational study of 63,257 participants in Singapore has also shown that incrementally higher red meat intake was strongly associated with increasingly higher risk of ESRD in a dose-dependent manner (HR (95% CI) for the highest vs. lowest quartiles of intake: 1.40 (1.15–1.71)) [37]. However, a secondary analysis of type 2 diabetic patients with proteinuria from the Ongoing-Telmisartan-Alone-and-in-combination-with-Ramipril-Global-Endpoint-Trial (ONTARGET) showed that higher animal protein intake was associated with a lower risk of CKD, albeit a non-significant difference. Furthermore, plant protein (defined as protein from tofu/soybean curd, legumes, and whole and refined grains) consumption was not found to be associated with CKD progression, although greater vegetable (leafy green, raw, and cooked vegetables) and fruit consumption was associated with lower risk of CKD progression [38].

Varying sources of dietary protein may also differentially impact CKD-related complications in DKD patients. For example, it has been suggested that higher consumption of vegetable protein sources among patients with advanced CKD may result in phosphate and potassium derangements. In a report of stage 3–4 CKD patients who were administered an omnivore diet containing 70% of protein from plant sources over a four-week period, lower phosphate, sodium, and titratable acid excretion in urine were observed; in contrast, there were no significant changes in serum phosphorus, parathyroid hormone, nor Fibroblast-Growth-Factor 23 level according to dietary protein source [39]. In addition, two episodes of mild hyperkalemia were reported which were corrected with food substitutions. Yet in a study of 14,866 participants from the NHANES III cohort, a higher proportion of dietary protein intake from plant sources (plant protein to total protein ratio) was associated with lower mortality in those with eGFR < 60 mL/min/1.73 m^2^, but not in those with eGFR ≥ 60 mL/min/1.73 m^2^ [40]. Future studies are needed to determine the effect of protein type upon kidney function and other relevant outcomes in the DKD population.

## 4. Dietary Protein Intake in Diabetic Kidney Disease Patients Receiving Dialysis

It should be highlighted that the aforementioned guidelines and studies do not apply to diabetic patients with ESRD receiving dialysis. Given that ESRD patients are predisposed to protein catabolism and losses of protein and amino acids in the dialysate [41], dietary protein restriction may lead to protein energy malnutrition (PEW), a potent predictor of mortality in this population [42]. Several observational studies have shown that low dietary protein intake ascertained by protein equivalent of nitrogen appearance normalized to body weight (nPNA) is associated with higher hospitalization and mortality risk in hemodialysis patients [43,44]. The National Kidney Foundation- Kidney Disease Outcomes Quality Initiative (NKF-KDOQI) Guidelines for Dialysis Patients indeed recommend higher dietary protein intake (>1.2 g/kg BW/day) as compared to non-dialysis dependent CKD patients [45]. While various markers of overnutrition, such as increased body mass index or high serum cholesterol levels, have shown deleterious effects upon cardiovascular disease and death in the general population, multiple observational studies have paradoxically shown survival benefit in dialysis patients [46,47]. Notably, in a Taiwanese study of 21 vegetarian dialysis patients and 42 age- and sex-matched non-vegetarian dialysis patients selected as controls, those who followed vegetarian diets demonstrated markers of subclinical protein malnutrition and vitamin D deficiency [48]. Indeed, monitored liberalization of protein intake is needed to ensure adequate dietary intake and prevention of PEW in diabetic ESRD patients receiving dialysis [49].

## 5. Energy and Carbohydrate Intake in Diabetic Kidney Disease

In the broader CKD population, including those who are non-dialysis and dialysis dependent, the NKF-KDOQI guidelines and the International Society of Renal Nutrition and Metabolism recommend a total energy intake of 30–35 kcal/kg BW/day which should be tailored to physical activity levels [45,50]. In elderly patients who are sedentary, energy intake of 30 kcal/kg BW/day may be sufficient. These recommendations apply to all non-dialysis and dialysis dependent CKD patients irrespective of their etiology of kidney disease (i.e., diabetic and non-diabetic kidney disease).

Avoidance of obesity is also an important strategy in preventing the development and progression of CKD. For example, observational studies have shown that obesity is associated with a higher incidence of CKD, and moderate weight reduction (5–10% of body weight) has been recommended in obese CKD patients to prevent kidney disease progression [51]. In addition to increased physical activity, reduction of caloric intake by 500–750 kcal/day or an intake of 1200–1500 kcal/day in women and 1500–1800 kcal/day in men is recommended in patients with type 2 diabetes of overweight or obese status [6,52]. However, a low carbohydrate diet that is replaced by high dietary protein sources should be avoided in DKD patients [53] given the harmful effects of a high protein diet on CKD trajectory, as described above. Experimental animal models have shown that, while low-carbohydrate, high-protein diets led to leaner body habitus in mice, those that received high-carbohydrate, low-protein diets experienced less illness, lower blood pressure, better glucose tolerance, lower cholesterol levels, and longer life span [54]. Observational data from NHANES has also demonstrated that diabetic participants with high protein intake (≥20% of total caloric intake) as assessed by dietary interview had higher risk of mortality compared to those with ≤10% of caloric intake from protein. These associations were attenuated if the source of proteins were plant-based [55]. However, another study demonstrated that high carbohydrate intake was associated with higher risk of CKD [38]. In conclusion, there remain substantial knowledge gaps with respect to the impact of carbohydrate intake upon outcomes in DKD patients [56], and future studies in this area are warranted. Given that ~45–60% of energy intake is obtained from carbohydrate sources [6], careful consideration should be made with respect to the source of dietary carbohydrates. For example, carbohydrates from sugars should be restricted to less than 10% of energy intake, and higher glycemic index foods should be substituted with low glycemic index foods such as whole grains, fiber, fresh fruit, and vegetables [7,8,12]. However, these types of food are often restricted in advanced stages of DKD given their high potassium and phosphorus content. Thus, consumption of fruits and vegetables with low potassium content, appropriate prescription of phosphorus binders, avoidance of processed convenience foods with high phosphorus content, and cooking procedures that reduce potassium and phosphorus levels in food are recommended [5].

## 6. Fat Intake in Diabetic Kidney Disease

There are multiple areas of uncertainty surrounding ideal dietary fat intake in DKD. [12] Although treatment of dyslipidemia is a critical aspect of the management of DKD patients given their high risk of cardiovascular disease and death [57,58], the optimal amount of dietary fat intake has not yet been defined. There is a general consensus across guidelines that a reduction of saturated fatty acids (SFA) and trans-fat intake contributes to a reduction in risk of cardiovascular disease in patients with diabetes [7,59]. Thus, it is typically recommended that saturated fats be limited to <7% of total daily calories [7,21,60]. Existing literature indicate that the type of fat consumed has a greater bearing upon metabolic status than total fat intake *per se*. Omega-3 and 6 polyunsaturated fatty acids (PUFAs) and monounsaturated fatty acids (MUFAs) were found to have beneficial impact upon DKD outcomes through the attenuation of inflammation and endothelial dysfunction and improved control of hypertension and dyslipidemia [61]. In a multicenter prospective study of 192 patients with type 1 and 2 diabetes and albuminuria, those who experienced a rise in albuminuria over time had higher SFA to PUFA ratios of dietary fat consumption after a seven-year follow up period [62]. Another secondary analysis of patients with type 1 diabetes from the Diabetes Control and Complication Trial (DCCT) showed that the absolute level of albuminuria was lower among those with greater long chain omega-3 PUFA consumption [63]. However, PUFA consumption did not impact the incidence of new onset albuminuria. A recent case-control study also observed a marginally significant inverse trend between higher dietary PUFA intake and lower incidence of ESRD [64]. These observations corroborate data from studies in the general population which have generally shown a protective effect of omega-3 upon cardiovascular outcomes [61]. However, it should be noted that the Outcome-Reduction-with-an-Initial-Glargine-Intervention (ORIGIN) trial of 12,537 patients with impaired fasting glucose, impaired glucose tolerance, or type 2 diabetes showed a negative effect of omega-3 PUFA upon cardiovascular disease and mortality [65].

## 7. Sodium Intake in Diabetic Kidney Disease

Prior data has shown that low dietary sodium intake is associated with reductions in blood pressure and proteinuria in CKD patients, which may be extrapolated to DKD patients [5,66]. Current nutritional guidelines for DKD patients uniformly recommend restriction of dietary sodium intake to less than 1.5–2.3 g/day (5 g of sodium chloride) [8,9,12]. However, some studies have reported that excessively low sodium intake adversely affects glucose metabolism and decreases insulin sensitivity. In addition, the activation of the renin–angiotensin–aldosterone system and sympathetic nervous system following low dietary sodium intake may further reduce insulin sensitivity [67]. At this time, sodium restriction should be individualized, and further research examining sodium intake thresholds and outcomes across various populations is needed.

## 8. Comparison of Prescribed Diets

An alternative approach in the management of chronic disease populations, such as those with diabetes, hypertension, and CKD, is to prescribe a comprehensive diet, such as the Dietary Approaches to Stop Hypertension (DASH) and Mediterranean diets, as opposed to focusing on individual nutrients. For example, both of the aforementioned diets emphasize a greater intake of vegetables, whole grains (i.e., complex and unrefined carbohydrates), and fruit and plant proteins (e.g., nuts, seeds, and beans). Compared to Western diets, they also have a lower proportion of animal protein and whole-fat dairy products. Studies of the DASH diet have suggested that it has a favorable impact upon blood pressure and the incidence of diabetes, although it is unknown as to whether this is due to its protein content or other components (e.g., potassium or isoflavones) [60,68,69]. In a study of 14,882 patients with an eGFR ≥ 60 mL/min/1.73 m^2^ from the Atherosclerosis-Risk-In-Communities (ARIC) cohort, the DASH diet was associated with lower risk of kidney disease after a median follow up of 23 years, independent of socio-demographics and baseline kidney function [70]. Most recently, a study of 1630 participants without underlying CKD from the Tehran Lipid and Glucose Study conducted food frequency questionnaire assessment with assignment of DASH-style diet scores based on these data [71,72]. After a mean follow up of six years, it was found that participants in the highest quintile of the DASH-style diet had a lower incidence of CKD compared to those in the lowest quintile. However, given that the DASH diet prescribes a higher level of dietary protein intake (~<1.4 g/kg BW/day), application of this diet to DKD patients with non-dialysis dependent CKD should be modified. An RCT of the DASH diet (containing 18% energy from protein) versus control diet administered over eight weeks did not show improvement in albuminuria, while reductions were seen in those who received a fruit and vegetable diet [73]. The high potassium (4.5 g/day) and phosphorus (1.7 g/day) content of the DASH diet may also limit its broad implementation in advanced CKD. Given that the DASH trial only enrolled participants with preserved renal function (eGFR ≥ 60 mL/min/1.73 m^2^ and serum creatinine <1.2 mg/dL), its safety and efficacy in those with moderate to advanced stages of CKD are unknown [60].

Compared to the DASH diet, characteristics of the Mediterranean diet include a high monounsaturated versus saturated fat ratio by using olive oil, as well as moderate red wine consumption. In spite of unrestricted fat consumption, many studies demonstrated that it resulted in a lower incidence of major cardiovascular events and type 2 diabetes [7,74,75]. However, there were no differences in all-cause mortality between the DASH versus control diets [76]. The Mediterranean diet has also been associated with lower risk of metabolic syndrome after kidney transplantation [77]. With respect to kidney disease outcomes, a 15-year observational study demonstrated that adherence to the Mediterranean diet was associated with lower risk of rapid decline in eGFR [78]. An RCT has also shown that the Mediterranean diet led to significant improvement in eGFR after two years. However, similar reno-protective effects were observed in those who received low carbohydrate and fat diets [79]. Further studies of the impact of the DASH and Mediterranean diets upon DKD outcomes are needed.

## 9. Practical Implementation of Nutritional Management

While adherence to nutritional guidelines may be challenging among DKD patients who bear multiple concurrent comorbidities resulting in complex medication regimens and recommendations from multiple providers, several strategies may be implemented that enhance its successful implementation. For example, data has shown that self-monitoring of food intake and feedback by clinicians can substantially improve adherence [80]. Frequent and clear communication with DKD patients about the importance of diet in their overall chronic disease management may further encourage adherence [81]. Using simplified diet approaches and instructions, as well as periodic monitoring of dietary intake with questionnaires administered by clinicians, particularly renal dietitians, may also augment compliance [82,83]. In an RCT of stage 3 CKD patients, a renal nutrition education program that included individual classes and hands-on sessions about food types and recipes improved adherence to dietary recommendations [84]. Obtaining patients’ feedback upon preferred food types and tolerability is also an indispensable aspect of successful nutritional management [85].

## 10. Future Areas for Research

There are multiple areas of uncertainty with respect to the nutritional management of DKD patients, including (1) the optimal amount of dietary protein intake among non-dialysis dependent CKD patients with diabetes, as well as (2) the comparative effects of animal vs. plant-based protein sources upon CKD and cardiovascular outcomes; (3) the preferred types and amounts of dietary fats in the DKD diet; (4) the role of complex carbohydrate foods upon DKD outcomes; (5) the ideal proportion of food types in DKD diets; and (6) the impact of “healthy diets” such as the DASH and Mediterranean diets upon outcomes in moderate to advanced CKD patients. It should also be noted that patients with advanced DKD progressing to end-stage renal disease may experience spontaneous resolution of hypoglycemia, normalization of glycated hemoglobin levels, and frequent hypoglycemic episodes necessitating discontinuation of anti-diabetic medications, known as the “burnt-out diabetes” phenomenon [86], and further studies in this population are particularly needed to determine the safety and efficacy of dietary restrictions in this population [49]. Given the exceedingly high morbidity and mortality of DKD patients, as well as compelling evidence demonstrating the critical importance of nutritional status in the broader CKD population, future studies that define the optimal nutritional management of this population may have a major impact upon improving the health and survival of this population.

## Figures and Tables

**Table 1:** Summary of dietary management of patients with diabetic kidney disease based on National Kidney Foundation Kidney Disease Outcomes and Quality Initiative and American Diabetes Association/National Kidney Foundation/American Society of Nephrology guidelines [8,12].

Nutrient	Guidance for Quantity	Guidance for Quality	Special Considerations
Protein	<15% of total calories, or RDA of 0.8 g/kg BW/day for patients with DKD.	Emphasize vegan protein sources, and non-fat or low-fat dairy products are recommended.	Modified to >1.2 g/kg BW/day in patients with ESRD treated with dialysis.
Carbohydrate	Specific recommendation was not provided.	Choice of high fiber fruits and vegetables. No more than 10% of daily calories as simple sugars.	Monitor potassium and phosphatelevels.
Fat	Specific recommendation was not provided.	Recommend omega-3 and omega-9 polyunsaturated fatty acid consumption.	Within meal plan for calories and palatability.
Sodium	1.5–2.3 g of sodium/day.	Use non-processed fresh food, and utilize sodium-free herbs and spices.	Sodium restrictions should be individualized.

Abbreviations: RDA, recommended dietary intake; BW, body weight; DKD, diabetic kidney disease; ESRD, end stage renal disease.
